# Mechanical Properties of Wooden Elements with 3D Printed Reinforcement from Polymers and Carbon

**DOI:** 10.3390/ma17061244

**Published:** 2024-03-08

**Authors:** Jan Dedek, David Juračka, David Bujdoš, Petr Lehner

**Affiliations:** 1 Department of Building Constructions, Faculty of Civil Engineering, VSB-Technical University of Ostrava, Ludvika Podeste 1875/17, 708 00 Ostrava, Czech Republic; 2 Department of Structural Mechanics, Faculty of Civil Engineering, VSB-Technical University of Ostrava, Ludvika Podeste 1875/17, 708 00 Ostrava, Czech Republic; 3 Department of Building Materials and Diagnostics of Structures, Faculty of Civil Engineering, VSB-Technical University of Ostrava, Ludvika Podeste 1875/17, 708 00 Ostrava, Czech Republic

**Keywords:** wood, mechanical properties, 3D print, carbon fibers, polymers, polycarbonate, lamellas

## Abstract

The research presented in this article aimed to investigate the differences in mechanical properties between solid structural timber and the same reinforced element in three different ways. A three-point bending test was performed on wood elements reinforced with carbon-fiber-reinforced polymer (CFRP), 3D printed polycarbonate (3DPC) lamellas, and 3D printed polycarbonate with carbon fiber (3DPCCF) lamellas. In this comparison, the bending strength was large for CFRP samples, which have 8% higher performance than samples with 3DPCCF and 19% higher performance than samples with 3DPC. Conversely, when factoring in theoretical manufacturing costs, the performance of 3DPCCF is almost three times that of CFRP and 3DPC. In addition, 3D materials can be used for more complicated reinforcement shapes than those discussed in the paper.

## 1. Introduction

Wood is one of the oldest building materials. Historically, it is the most widely used material for roof and ceiling construction. Unfortunately, it is very susceptible to failure; if a timber element is incorrectly incorporated into a structure or if its functioning conditions are adversely affected during reconstruction and restoration, the timber can suffer from the effects of moisture with subsequent infestation by woodboring pests, causing a significant reduction in the load bearing capacity of structural timber elements [1,2,3,4]. Ceiling beams, rafters, purlins, and battens are subjected to tensile stress and compressive strength due to bending, so materials with good tensile strength are mainly used for reinforcement on the bottom. Conventionally, strip steel or rolled steel sections have been used for the reinforcement of timber elements, but their shape and visibility can alter the overall architectural character of the structure being restored. Nowadays, a more suitable variant of reinforcement of wooden elements is the use of composite lamellas made of carbon fiber and epoxy resin, which no longer interfere to such an extent with the spatial character of the structure, but are significantly limited in shape and color [5,6].

The use of carbon-fiber-reinforced polymer (CFRP) lamellas has been studied in detail in several publications [7]. In further studies, an example of the different behavior in fire [8] or the problem of the significant difference in brittleness between wood and carbon reinforcement is analyzed [9].

Similarly, 3D printing is spreading to all sectors, and this is also true for construction [10,11,12,13]. Different materials suitable for 3D printing were explored, as well as the printing methods themselves [14]. The 3D printing technology of cementitious materials has a significant contribution to the future, but there are other research tasks [15]. There is great interest in the use of 3D printing in the application of wood-based material in combination with polymers and other materials [16], which leads to greater sustainability. Apart from the actual use of 3D printing for interesting shapes of structural elements [17], the combination with wood is an interesting way [18]. The authors have shown interesting results by combining complex structures; however, the goal of the work was to use 3D printing as quickly as possible and apply it to an existing structure. Therefore, the use of the lamella shape was approached. Lamellas printed using 3D printing can not only improve the mechanical properties of the wooden element but also approach it in shape, color, and texture and not interfere so significantly with the architectural character of the rehabilitated structure.

This research aimed to determine the differences in mechanical properties between solid structural timber and the same reinforced element in three different ways. In addition to the already-known use of CFRP, the possibility of applying 3D printed lamellas made of polycarbonate and polycarbonate with carbon fiber admixture is presented. The experiment was prepared on the basis of a three-point bending test. The present study provides an extended view of the applicability of 3D printing for repairing wooden structures or for designing new structures combining nontraditional material pairs. The results of the performance of the same test for different combinations provide clear evidence of the continued need for research in this area. The article contains the materials and methodology used, as well as the results and their evaluation.

## 2. Materials and Methods

The purpose of the research was to determine the differences between different approaches to the reinforcement of a wooden element in the same experiment. The properties of the different materials, the preparation of the test specimens, and the description of the experiment and evaluation are presented here.

### 2.1. Wood

**Figure 1:**
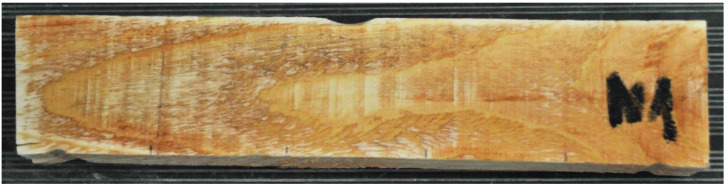
Example of a wooden prism (after experiment).

Due to the goal of using lamellas to repair, for example, timber elements such as pitched roof cladding, standard elements of 30 × 50 mm cross-section with a guaranteed class C30 timber (according to EN 338 [19]) were purchased (see Figure 1). The type of wood was spruce. This timber has the specified properties according to the standard at the level shown in Table 1. Wood is a very inhomogeneous material with a large variance of properties. This fact must be taken into account when evaluating the measurement results.

### 2.2. Carbon-Fiber-Reinforced Polymer Lamellas

A CFRP lamella, which is standardly used for the reinforcement of various building structures and materials [20], was chosen as the first reinforcement element. The SANAX CFRP lamella (CarboLamela, Sanax chemical construction s.r.o., Decin, Czech Republic) [21], which contains at least 68% carbon fiber, was used. The lamellas are made of carefully aligned carbon fibers bonded with a synthetic resin.

**Figure 2:**
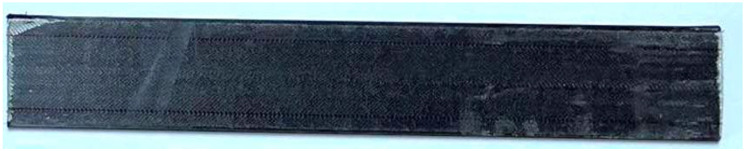
Example of a CFRP lamella.

According to the manufacturer, the fiber content is 68% and the resin content is 32% by volume. The properties of the CFRP lamella are given in Table 2. This lamella is mainly used to reinforce concrete structures. For the research presented, a unidirectional lamella of 30 × 230 mm with a thickness of 1.4 mm was chosen (see Figure 2). The lamella was purchased in a 2000 mm roll and cut to the required length using a saw.

### 2.3. Three-Dimensional Printed Lamellas—Polycarbonate Blend

**Figure 3:**
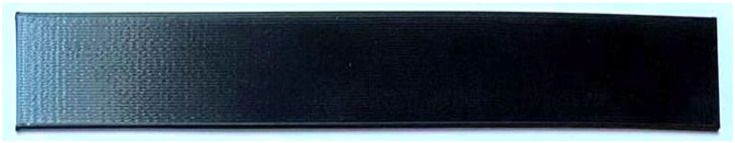
Example of a PC blend lamella.

As an alternative to the carbon lamella, a sample printed on a 3D printer directly was chosen. One of the strongest materials for this type of printer (FFF/FDM), a polycarbonate (PC) blend, was selected for production. The samples were processed on the Prusa i3 MK3S+ 3D printer (Prusa Research, Praha, Czech Republic) [22] with PrusaSlicer 2.7.0 preparation software [23]. The printed filament before processing had a diameter of 1.75 mm. During 3D printing, it was fused to the printer head and reflected in the nozzle dimension. The nozzle temperature was 215 °C, and the printing speed was 60 mm/s. In this case, the samples were printed with 0.4 × 0.2 mm cross-sectional filament. Thus, the filaments are all aligned in the longitudinal direction, fully simulating the distribution of fibers in wood as the original material. The fibers are stretched when subjected to bending tensile stress. The size and direction of the filament used in the printing and the effect on mechanical properties have already been investigated and have shown relatively small differences [17]. Moreover, the complexity of printing to cross is not advantageous compared to the result. Therefore, the printing of the lamellas longitudinally in all layers was used (see Figure 3). The properties of the material are shown in Table 3. There is a clear difference in material properties compared to CFRP material.

### 2.4. Three-Dimensional Printed Lamellas—Polycarbonate Blend Carbon Fibers

**Figure 4:**
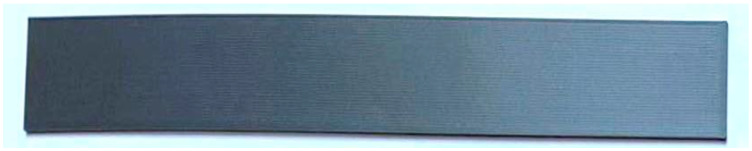
Example of PC blend carbon fiber lamella.

As the last alternative for reinforcement, PC blend carbon fiber 3D filament was chosen. Unlike the PC blend, this has higher demands on the printer itself, as carbon fiber increases the fragility of the raw material. On the contrary, it has better durability and different mechanical and thermal parameters. The properties are listed in Table 4, and the printed lamella is shown in Figure 4. The preparation of the printed lamella is identical to that of the previous material.

### 2.5. Sample Preparation

The wooden prismatic samples were cut to the length required for the planned experiment. Due to the limited length of the 3D printing, the length of the test body was set to 230 mm only. In total, 20 test bodies were created.

The first 5 test samples (No. N1–N5—“N” means not-reinforcement) were not reinforced; these samples were used to determine the characteristic bending strength of the element.

**Figure 5:**
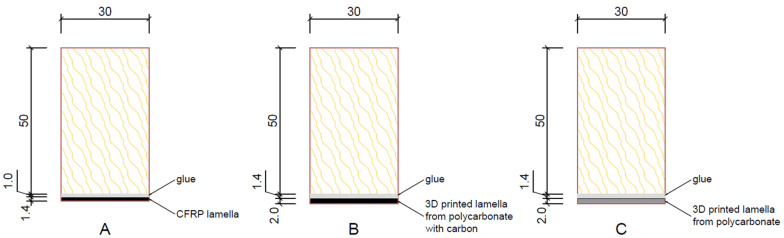
Samples: (**A**) wooden element with CFRP lamella, (**B**) wooden element with 3D printed lamella from polycarbonate with carbon fibers, (**C**) wooden element with 3D printed lamella from polycarbonate.

The next 5 test pieces (No. L1–L5) were reinforced with CFRP (see Figure 5A) A two-component epoxy adhesive, Carboresin [21], was used to bond the lamella to the test piece and was applied with a squeegee with a full thickness of approximately 1.0 mm.

The other 5 test samples (No. L6–L10) were reinforced with a printed PC blend carbon fiber lamella—again the lamella size was 30 × 230 mm, but the lamella thickness was 2.0 mm. The larger thickness was used due to the nozzle on the 3D printer not being able to print a lamella of the same thickness as the CFRP carbon fiber lamella mentioned above (Figure 5B).

The last 5 test samples (No. L11–L15) were reinforced with a printed lamella made of the PC blend, a polycarbonate blend material (Figure 5C). The size and thickness of the lamella were the same as those of the previously printed lamella, i.e., 30 × 230 mm with a thickness of 2.0 mm. The printed lamellas were again adhered all over, and the adhesive thickness was approximately 1.4 mm. The difference in reinforcement thicknesses is evaluated in the results.

### 2.6. Three-Point Bending Test

**Figure 6:**
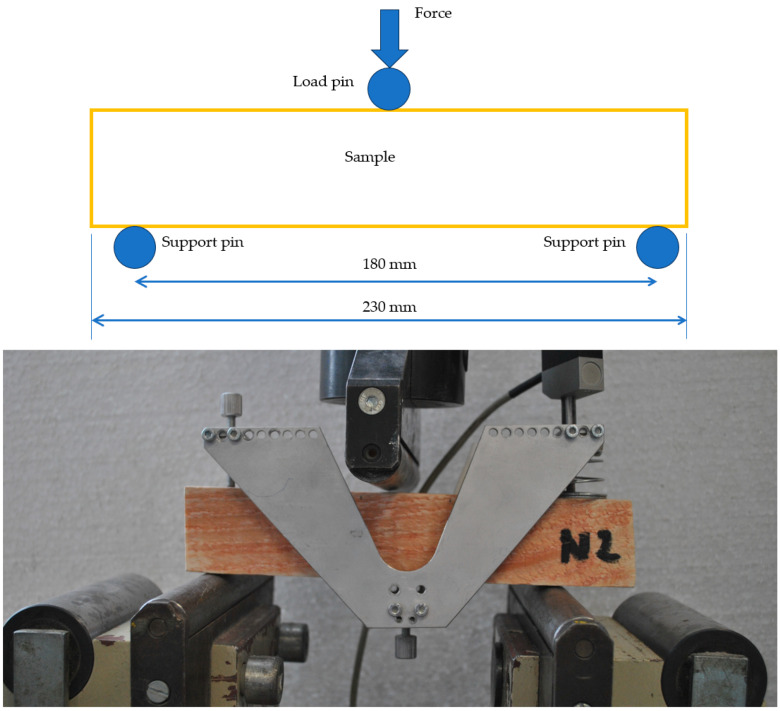
Wood sample in the machine before the experiment (**bottom**), scheme of the test (**top**).

The three-point bending test based on ASTM D143-22 is one of the basic experiments carried out on building materials [24]. The test was carried out on a universal test machine according to the scheme and photography in Figure 6. It was Formtest 300 kN (Seidner & Co. GmbH, Riedlingen, Germeny). The span of the supports was 180 mm. A displacement sensor was placed directly under the load panel. The force and deflection were recorded on the machine. These values were then plotted on a graph, and other properties were calculated.

### 2.7. Simple Cost Calculation

All tested samples contain raw wood and glue. These parts were not included in the calculation. All materials were purchased from the Czech Republic. For costing purposes, the cost per CFRP lamellas sample was calculated for simplicity. Two meters of CFRP lamella cost approximately EUR 35, so one sample for testing costs approximately EUR 4. In addition, the printed samples were created on the 3D printer itself, but taking into account material, energy, and other criteria, it can be assumed that one PC blend lamella costs approximately EUR 1.20 and one PC blend carbon fiber lamella costs approximately EUR 1.60. It should be said that future commercial developments would reduce the cost of 3D printing even further.

## 3. Results and Discussion

The evaluation of the laboratory tests was carried out in several steps. First, the data from the testing machine, represented as force–deformation diagrams, were processed for each group of samples. The maximum forces achieved before the first failure were then determined. For each set, five values were obtained from which the mean and coefficient of variation (CV) were calculated. The higher variability is defined by the name of the data.

Furthermore, because the CFRP lamellas were 1.4 mm thick and the 3D lamellas were 2.0 mm thick, the results of the maximum forces achieved were converted by the bending moment to the bending strength. In the next step, the price categories presented in Section 2.7 were included by increasing the adjusted values from the PC blend and PC blend carbon fiber samples by a multiple of 3.3 and 2.5, respectively, which is the ratio between the material preparation prices.

### 3.1. Force–Deformation Diagrams

**Figure 7:**
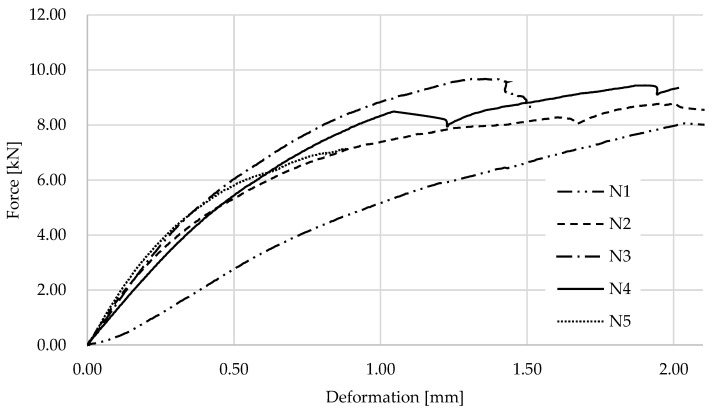
Results from an experiment on solid wood samples.

**Figure 8:**
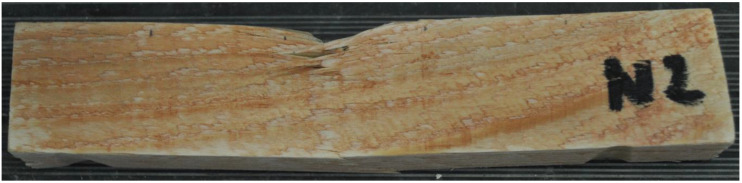
Solid wood sample after experiment.

From the measurements of all samples, the loading force values over time and the corresponding deformation were recorded. The graphs in Figure 7 present the results for wooden samples. A higher dispersion of the results was observed, which is common for wooden materials. Four samples showed similar behavior and graph progression during the test. One sample shows a different graph shape. However, the maximum is not significantly different. Figure 8 shows one of the samples after the test. The failure occurred in the load head region and in the lower tensile region.

**Figure 9:**
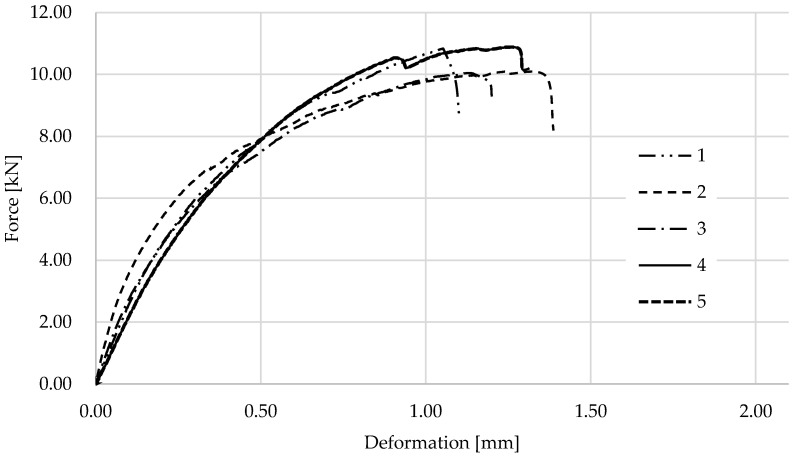
Results of an experiment in wood with CFRP samples.

**Figure 10:**
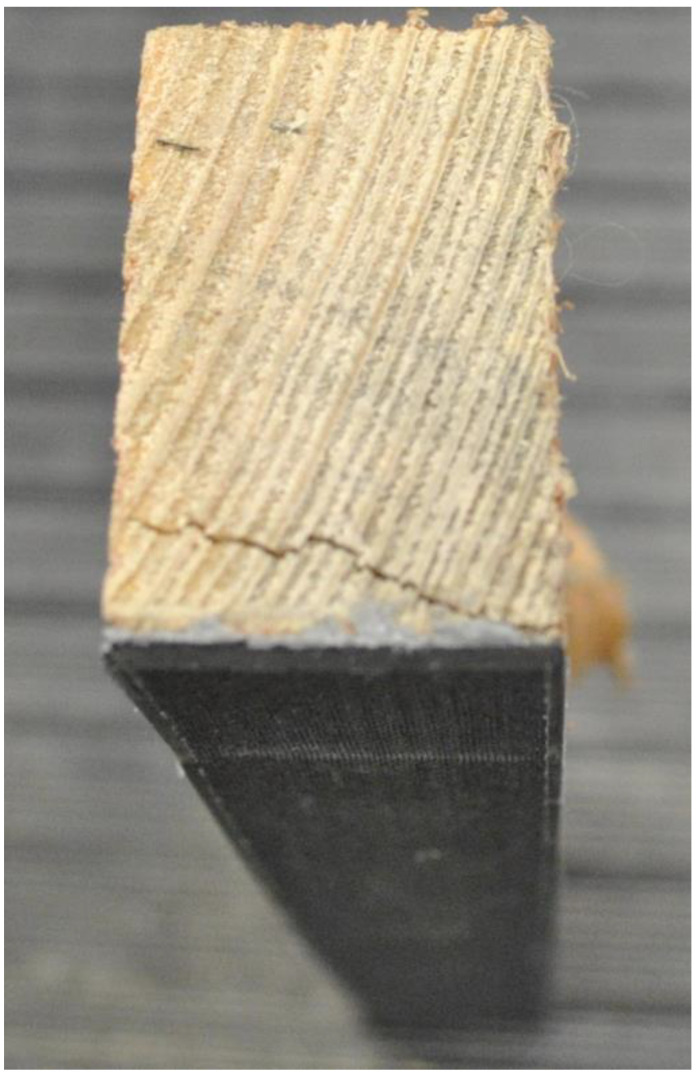
Side view of the CFRP sample after the experiment.

The graphs in Figure 9 show the results for the wooden samples with CFRP. All tests showed similar behavior and progression. For several of the graphs, there is a jump at the peaks where the wood failure occurred, and the load-carrying capacity was taken over by the CFRP lamella. Figure 10 shows one of the specimens after testing from a side view, where there is a very clear failure in the wood starting at the lamella connection interface.

**Figure 11:**
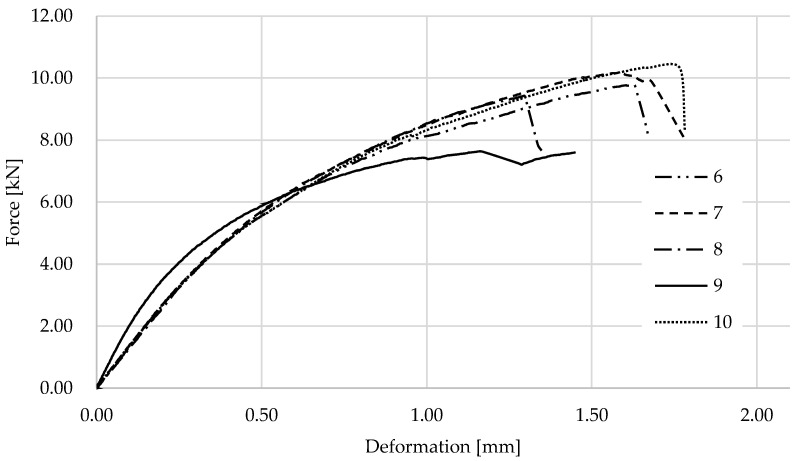
Results of the experiment on wood with PC blend carbon fiber samples.

**Figure 12:**
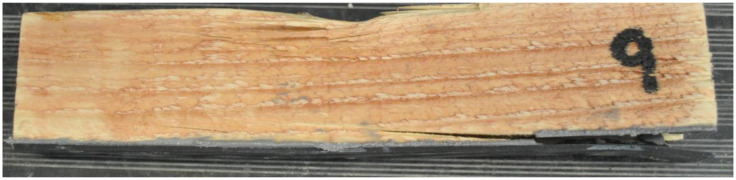
View of the PC blend carbon fiber sample after the experiment.

The graphs in Figure 11 show the results for wooden samples with 3D printed PC blend carbon fiber lamellas. Most tests were performed similarly, with only one sample failing at a lower force value. Figure 12 shows one of the specimens after testing, where the failure of the wood and the printed lamella is very clear. As expected, the PC blend carbon fiber lamellas are more brittle than the other samples.

**Figure 13:**
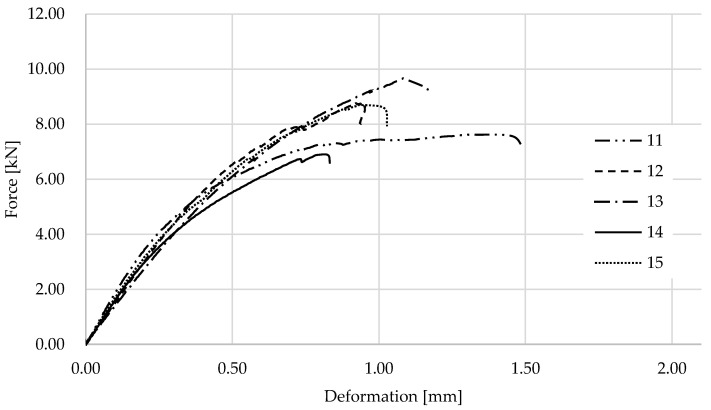
Results of an experiment on wood with PC blend samples.

**Figure 14:**
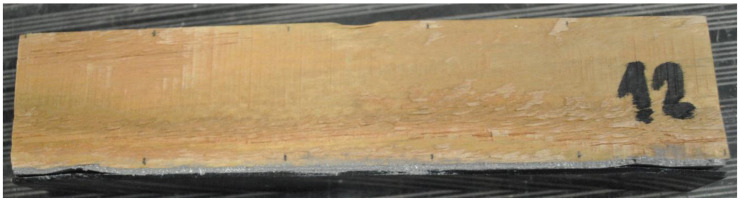
View of the PC blend sample after the experiment.

The graphs in Figure 13 show the results for wooden samples with 3D printed PC blend lamellas. The samples failed at less deformation than the other groups of samples. Figure 14 shows one of the specimens after testing, where it is very clear that the lamellas on the supports were pushed through and that the connection to the wood failed.

### 3.2. Statistical Evaluation

The maximum forces achieved were subtracted from all graphs. Then, the means and coefficient of variation were calculated for each group. Table 5 shows the results of the evaluation. The table shows five values for each group, the average of the results, and the coefficient of variation calculated from the standard deviation of the measured group. Data from the test samples with PC blend lamellas have a higher coefficient of variation than the solid and CFRP samples. Since CFRP has a much higher strength than wood, it can be expected that wood with CFRP will have a beneficial effect on the durability of the specimens. The results of CFRP samples are about 21% higher than those of solid wood, and the results of PC blend carbon fiber samples are about 12% higher than those of solid wood. The samples with the PC blend are on average on par with solid wood and did not show an increase in load-carrying capacity. However, this fact should be taken into account for further possibilities of increasing the amount of material, changing the shape of the 3D printing, and other possible improvements. Furthermore, it is interesting from the perspective that the raw PC blend and PC blend carbon fiber have almost the same tensile strength. In contrast, the high tensile strength of raw CFRP is different. A further evaluation of performance as a function of material quantity and price is presented in the next section.

### 3.3. Evaluation

The maximum forces obtained from each group were converted to bending strength. Previously, the maximum moment of the static system and the modulus of the bending section were determined. Table 6 shows the results. The comparison of maximum strength is best for the CFRP samples, which achieved 10% higher values than the 3D PC CB and 21% higher values than the 3D PC in the test. Looking at flexural strength values, CFRP results are 8% higher compared to 3D PCCB and 19% higher than 3D PC values. On the other hand, the recalculation including costs gives a different view of the results. The highest values are achieved by the 3D PC blend specimens and are even almost triple those achieved by CFRP. Also, 3D PC blend carbon fiber has a large value.

## 4. Conclusions

As a result of the research, the mechanical properties of a part of a solid wood structure and a part of a reinforced wood structure in different variants were investigated. A three-point bending test was carried out on wooden members reinforced with carbon-fiber-reinforced polymer, 3D printed polycarbonate, and 3D printed carbon-fiber-reinforced polycarbonate. In this comparison, the bending strength was large for CFRP samples, which have 8% higher performance than samples with 3DPCCF and 19% higher performance than samples with 3DPC. Conversely, when factoring in theoretical manufacturing costs, the performance of 3DPCCF is almost three times that of CFRP and 3DPC. In addition, 3D materials can be used for more complicated reinforcement shapes than those discussed in the paper. The findings can be used for further research to improve the sustainability of future and existing building structures when they are repaired. Three-dimensional printing itself is undergoing very rapid development, opening up great possibilities for further application in the direction outlined here.

## Figures and Tables

**Table 1:** Characteristic values for wood class C30.

Properties		Sign	Value
Strength Properties [MPa]	Bending	*f_m_*_,*k*_	30
	Tension parallel	*f_t_*_,0,*k*_	19
	Tension perpendicular	*f_t_*_,90,*k*_	0.4
	Compression parallel	*f_c_*_,__0,*k*_	24
	Compression perpendicular	*f_c_*_,__90,*k*_	2.7
	Shear	*f_v_*_,*k*_	4.0
Stiffness Properties [GPa]	Mean modulus of elasticity parallel	*E*_0,*mean*_	12.0
	5% modulus of elasticity parallel	*E*_0.05_	8.0
	Mean modulus of elasticity perpendicular	*E*_90,*mean*_	0.4
	Mean shear modulus	*G_mean_*	0.75
Density [kg/m^3^]		*ρ*	460

**Table 2:** Characteristic values for CFRP CarboLamela [21].

Properties	Sign	Value
Modulus of elasticity in tension [GPa]	*E*	170
Tensile strength [MPa]	*F_tu_*	3100
Proportional elongation at crack [%]	*ε_p_*	1.6–1.9
Fiber content [%]	-	68
Density [kg/m^3^]	ρ	1600

**Table 3:** Characteristic values for PC blend [23].

Properties	Sign	Value
Modulus of elasticity in tension [GPa]	*E*	1.9
Tensile strength [MPa]	*F_tu_*	63
Density [kg/m^3^]	ρ	1220

**Table 4:** Characteristic values for PC blend carbon fiber [23].

Properties	Sign	Value
Modulus of elasticity in tension [GPa]	*E*	3.5
Tensile strength [MPa]	*F_tu_*	64
Density [kg/m^3^]	ρ	1220

**Table 5:** Results of experiments.

Solid Wood		Wood with CFRP		Wood with 3D Printed PC Blend Carbon		Wood with 3D Printed PC Blend	
Mark	Maximum Force [kN]	Mark	Maximum Force [kN]	Mark	Maximum Force [kN]	Mark	Maximum Force [kN]
N1	8.062	L1	10.832	N1	8.062	L1	10.832
N2	8.277	L2	10.094	N2	8.277	L2	10.094
N3	7.475	L3	10.046	N3	7.475	L3	10.046
N4	9.673	L4	10.88	N4	9.673	L4	10.88
N5	8.491	L5	10.88	N5	8.491	L5	10.88
**Mean**	**8.396**	**Mean**	**10.5464**	**Mean**	**9.4796**	**Mean**	**8.3258**
**CV**	**0.086**	**CV**	**0.037**	**CV**	**0.104**	**CV**	**0.116**

**Table 6:** Results from experiments and after performance evaluation.

Type of Sample	Mean Force [kN]	Bending Strength [MPa]	Price Consideration Bending Strength [MPa/EUR]
CFRP	10.546	35.10	35.10
3D PC CF	9.480	32.29	80.73
3D PC	8.326	28.36	94.54

## Data Availability

Data are contained within the article.
